# Small Variations in Early-Life Environment Can Affect Coping Behaviour in Response to Foraging Challenge in the Three-Spined Stickleback

**DOI:** 10.1371/journal.pone.0147000

**Published:** 2016-02-10

**Authors:** M. Rohaa Langenhof, Rienk Apperloo, Jan Komdeur

**Affiliations:** Behavioural and Physiological Ecology, Groningen Institute for Evolutionary Life Sciences, University of Groningen, Nijenborgh 7, 9747 AG, Groningen, The Netherlands; University of Missouri, UNITED STATES

## Abstract

**Context:**

An increasing concern in the face of human expansion throughout natural habitats is whether animal populations can respond adaptively when confronted with challenges like environmental change and novelty. Behavioural flexibility is an important factor in estimating the adaptive potential of both individuals and populations, and predicting the degree to which they can cope with change.

**Study Design:**

This study on the three-spined stickleback (*Gasterosteus aculeatus*) is an empiric illustration of the degree of behavioural variation that can emerge between semi-natural systems within only a single generation. Wild-caught adult sticklebacks (P, N = 400) were randomly distributed in equal densities over 20 standardized semi-natural environments (ponds), and one year later offspring (F1, N = 652) were presented with repeated behavioural assays. Individuals were challenged to reach a food source through a novel transparent obstacle, during which exploration, activity, foraging, sociability and wall-biting behaviours were recorded through video observation. We found that coping responses of individuals from the first generation to this unfamiliar foraging challenge were related to even relatively small, naturally diversified variation in developmental environment. All measured behaviours were correlated with each other. Especially exploration, sociability and wall-biting were found to differ significantly between ponds. These differences could not be explained by stickleback density or the turbidity of the water.

**Findings:**

Our findings show that a) differences in early-life environment appear to affect stickleback feeding behaviour later in life; b) this is the case even when the environmental differences are only small, within natural parameters and diversified gradually; and c) effects are present despite semi-natural conditions that fluctuate during the year. Therefore, in behaviourally plastic animals like the stickleback, the adaptive response to human-induced habitat disturbance may occur rapidly (within one generation) and vary strongly based on the system’s (starting) conditions. This has important implications for the variability in animal behaviour, which may be much larger than expected from studying laboratory systems, as well as for the validity of predictions of population responses to change.

## Introduction

Animals are very sensitive to their environment [1]. For example, most animals are keenly aware of changes in daylight [2], temperature [3], and social dynamics within their population [4]. Sensitivity to the surrounding world confers great evolutionary advantages, as many aspects of population structure and species ecology are constrained by the environment [5]. Being able to predict, perceive and adequately respond to current environmental events, such as seasonality, food peaks and predation pressures, often directly affects the adaptive potential of animals [6,7][8].

In addition to being affected by their current surroundings, animals are often influenced by the circumstances they experienced during or shortly after birth. This phenomenon is commonly referred to as developmental plasticity [9]. The habitat that animals experience in their early life has a strong influence on both their physiological development, such as growth rate and the development of physical traits [10], and their behavioural development, such as the strategies they deploy later in life [9,11]. The early-life environment is not limited to physical characteristics such as food availability and territory quality, but can be convincingly argued to also include the social environment, as illustrated by several studies showing effects of the social group on later-life personality aspects [12,13]. For example, early-life exposure of certain fish to the risk of predation can increase somatic growth at juvenile stages and reduce size at adulthood [14]. Similarly, females of some bird species respond to intense mate competition by influencing the developmental pathways of their offspring in order to make them more competitive and/or aggressive [15]. Studies on epigenetics, which for example have shown the switching on and off of specific genes coding for specific behaviours induced by external or environmental factors, are beginning to understand the neural and molecular mechanisms behind these processes from studies in many animal and human models [16,17]. The importance of the early-life environment to not only the development of many aspects of adult functioning, but even on evolutionary processes themselves, has recently become more recognised [18].

Drastic changes in natural environments over the last few decades have challenged animals to respond to their environments with more flexibility [10,19–22]. Such changes include habitat fragmentation [23], chemical pollution [24] and the introduction of man-made structures like buildings, pipelines, or even simply trash. Instinctive behaviours that provided individuals with an advantage in the evolutionary past may suddenly provide a disadvantage when the environment does not meet expected norms. As such, the ability to alter behavioural or life-history traits in response to the conditions they experience, is fundamental to a population’s ability to deal with short-term environmental change [25].

When studying the responses of animals to human-induced environmental disturbance, the most commonly studied factors are at the physiological level, such as tolerance to chemicals, or at the population level, such as mortality and birth rates. However, the first response to environmental disturbances happens on a behavioural level, either by avoiding the disturbance through migration, or by finding a way to cope with it [22]. Animal adaptive responses at this level have been poorly studied, and have mostly been conducted in artificial laboratory settings rather than in more natural environments [26]). Especially foraging and mating behaviours, both of which directly impact fitness, are important in understanding the abilities of animals to cope with human-induced modification of their environment [27]. In addition, although large human-induced environmental changes have been studied extensively [22], the effect of small changes, especially during development, is not usually studied.

In this study, we investigated how differentially reared three-spined sticklebacks (*Gasterosteus oculeatus)*, a well-studied model species for behavioural plasticity and adaptive potential [28], behaved in response to human-induced disturbance of foraging, through presenting them with a novel object during scheduled feeding times. We observed five behaviours: exploration, activity, foraging, sociability and wall biting (see Table 1). Exploration, activity, and sociability are commonly used in animal personality studies [29,30]. Foraging relates to ability to utilise the environment and obtain resources. Wall biting is often considered a measure of stress [31]. Specifically, we asked 1) if individuals that were reared under different circumstances respond in statistically comparable ways; 2) if correlations between behaviours differ depending on rearing environment; and if so, 3) if such differences in suits of behaviours can be attributed to differences in group size or turbidity; 4) if sticklebacks performed differently over time through habituation and learning.

**Table 1:** Behaviours recorded for the first ten sticklebacks that entered the experimental setup, for 120–180 seconds. All measurements were corrected for observation time.

	Description	Unit
**Exploration**	The time (s) until successful manoeuvre through the novel obstacle represents exploration speed [30].	Seconds from start
**Activity**	The number of switches between spatial locations (cup, middle and wall, see Fig 1) recorded on 10 second intervals reflects habitat use and activity in an unfamiliar environment [30].	# spatial switches
**Foraging**	The number of bites at the food cup or the ground, both of which qualified as opportunity to find food, reflects the effort invested in foraging [51,52].	# bites at food
**Sociability**	The number of conspecifics within a body length averaged across recordings on 10 second intervals. As manoeuvring space was available, this reflects willingness to be near conspecifics [38].	# surrounding fish
**Wall biting**	The number of intervals at which the individual was found biting towards the transparent wall, which may be considered a measure of the amount of time spent in stress over the obstruction [34].	# intervals biting

These questions are interesting especially as this study utilised semi-natural environments, which allowed for observation of animals in a natural setting and in the presence of their own social group and habitat, and as it investigated relatively small ecological differences such as commonly occur between natural habitats, rather than large ecological differences such as the absence or presence of predation.

Regarding the first question, we expected that, influenced by a variety of environmental conditions including not only physical and nutritional differences but also maternal effects [17], social status [10] and early life stress [32], individuals inhabiting different environments would react differently to environmental novelty. Such effects have been shown previously in relation to environmental differences in frequency and magnitude of stressors [33]. If early-life experience and environment did not affect response to novelty, individuals from all environments would be expected to display comparable behaviours. Behavioural correlations, closely related to the increasingly popular concept of animal personality [34], have previously been shown to form in populations with a history of predation but not in predator-naive populations [35,36]. However, it is not clear whether these correlations can be generated within a single generation (i.e. without selection), and whether mild environmental differences in the absence of predation can trigger behavioural syndromes. In this study we investigate whether behavioural correlations can be generated within a single generation, and if so, whether such behavioural correlations are triggered by small differences in the rearing environment. Regarding the second question, if correlations between behaviours differ depending on rearing environment, we expected that stronger selective pressures than presented in this experiment would be required to induce differences in behavioural correlations between populations, as to date only actual predation has been successfully related to behavioural syndromes [35–37]. Regarding the third question, we expected that both group size and turbidity may be important factors in influencing development of foraging behaviours. The presence of conspecifics has been shown relevant for growth and behaviour in sticklebacks [13,38], and although some recent studies in other species found no effects of family or group size on exploration [39], group size is likely to affect both social learning and competition in a semi-natural setting. Turbidity affects visibility, sneaking success [40] and use of compensatory olfactory senses [41], and therefore may be a confounding factor in identifying and responding to a novel object. As nests in dense vegetation (i.e. high turbidity) have been found to have a higher egg survival rate than exposed ones [42], group size and turbidity may be related to each other.

This study adds to an existing body of literature exploring the effects of early-life environment on later-life behavioural responses, especially because it investigates behaviour under more natural conditions than most previous work, and may help to understand how small variations in habitat during the developmental period can affect the responses of animals to environmental disturbance. In terms of practical applications, the results of this study may be of use in predicting the reliability of models in conservation biology, whose accuracy often depends on estimates of environmental parameters [43,44].

## Methods

### 2.1 Population origins

**Fig 1:**
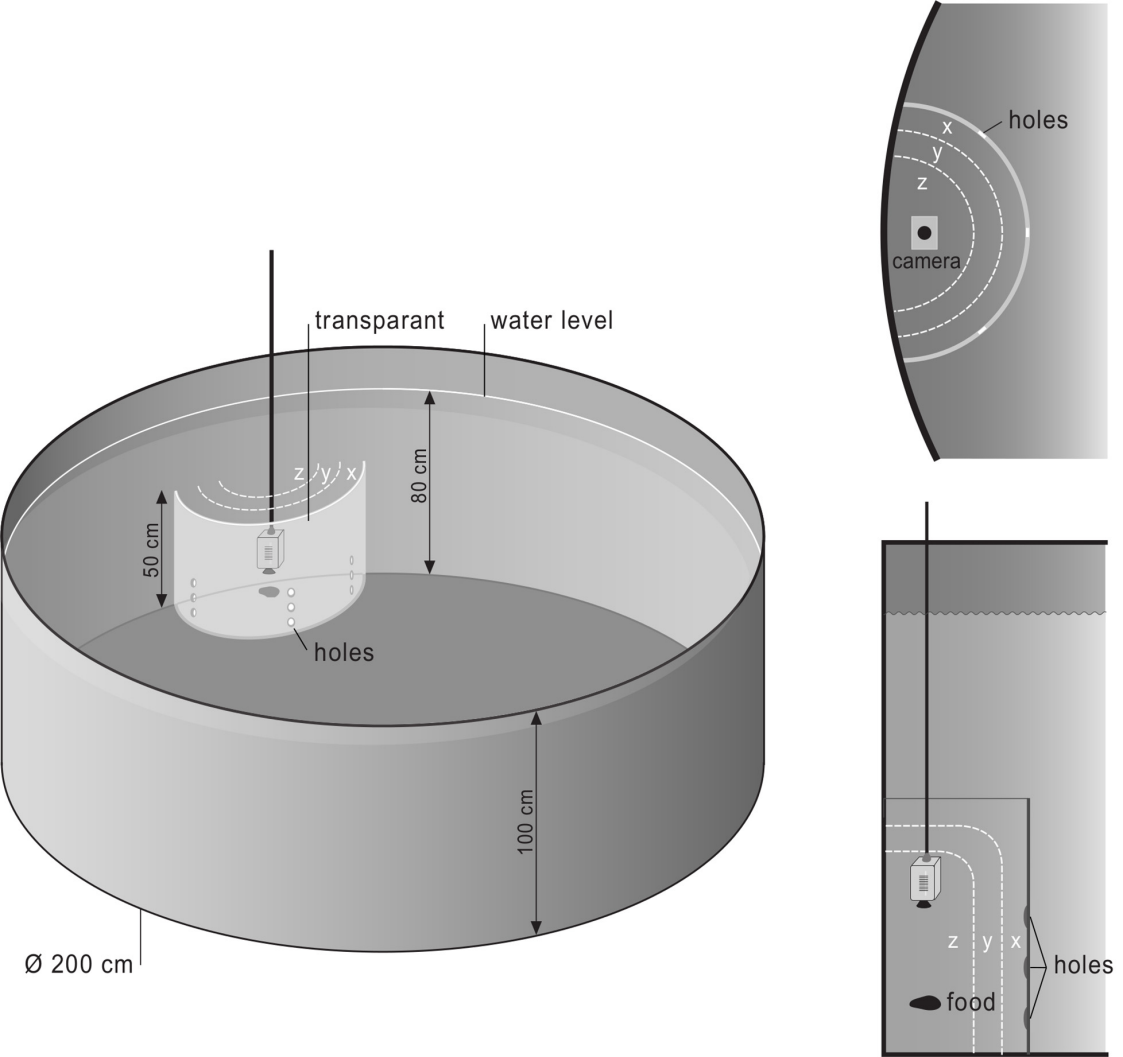
Layout of the experimental set-up. A: overview of the entire pond with the experimental setup against the wall; B: top view, where letters x, y, and z correspond to the wand, mid and cup zones respectively; C: side view.

Parents of the F1 generation sticklebacks used in this study were imported from a natural population in a privately owned pond named Cae Mawr on the isle of Anglesey, Wales (UK) in 2011. This population has been previously studied by [35] for its fifty-year-long history of non-predation and non-disturbance, and represents an excellent model system to research behavioural plasticity [28]. A total of 400 adults (P) were caught overnight with minnow traps for three consecutive days (in April 2011) and then transported to the Netherlands by car. Immediately upon arrival they were randomly distributed in equal densities over 20 small, semi-natural ponds at the University of Groningen (the Netherlands) (see Fig 1). In each of these ponds, offspring (F1) were born and naturally reared by their fathers, where they lived with 40–85 conspecifics depending on the breeding success of the 20 initial wild-caught sticklebacks placed in each pond. Care was taken to randomise placement of wild animals, to avoid bias of behavioural types (bold animals tend to be caught first).

### 2.2 Semi-natural setup

The experimental setup consisted of 20 semi-natural ponds of 200cm in diameter and 90cm in depth, surrounded by a netted enclosure to keep out predators (Fig 1A). Ponds were placed in a 2x10 grid, in order to equalise edge effects. There were no surrounding trees or buildings. A 10 cm edge around the ponds limited disturbance from the immediate surroundings and provided a buffer for rain water. Ponds were professionally cleaned to standardise pre-experimental conditions, filled with tap water and *Velda Bio-fit* pond chemicals to stabilise the water quality, and subsequently filled with 10 cm of small gravel stones. All ponds were provided with an oxygen bubbler and several habitat enrichments, consisting of PVC hideouts for cover and tall plastic plants for spatial partitioning.

As ponds diversified during a warm summer period just after breeding season, young individuals in each pond experienced varying conditions in important ecological criteria like fish density, turbidity and habitat history. It is worth noting that conditions in the ponds fluctuated during the year, and as such, F1 sticklebacks experienced naturally changing conditions in density, weather, water temperature, and algae cover including freezing conditions in winter. Natural diversification created ponds with differing degrees of algae growth (0.05–0.15% light let through) and stickleback population density (40–85 individuals at time of behavioural assays, average 64) within months. Turbidity was measured with a spectrophotometer. Algae growth limited visibility in some ponds, and 8 out of 20 ponds were excluded from experiments as visibility was too low to count pond density or accurately observe fish behaviour. In April 2012, population densities for each pond were estimated by averaging visual estimates at feeding time from 12 different naïve observers. Sticklebacks live 1–2 years on average [45], with wild animals having a shorter lifespan than lab-housed animals, and while we cannot be certain that all P-generation sticklebacks died a year after capture, based on length measurements from video recordings we estimate no more than 10% wild sticklebacks per pond at the time of the density estimates. No P-generation sticklebacks were identified during behavioural assays. Although we measured density and turbidity, we make no claim as to the exact nature of the environmental differences between ponds; ponds exhibited small and difficult-to-quantify variations in territory quality, nutrition, competition pressures, social structure, and visibility, but determining the relative impact of these parameters on the development of behaviour falls outside the scope of this study.

As consequence of the experimental choice to use semi-natural conditions, disturbances in the animal’s environment and social group have been kept to a minimum, which precluded us from marking individuals for identification. Marking sub-adults with fluorescent paint just under the stickles is a commonly used method in laboratory fish [14], but tends to result in a 10–20% mortality rate and presents a significant disturbance in both developmental processes and group cohesion. In addition, the stress from such a capture and recovery would impact the behavioural strategies used by the fish in the experimental setup, negating the advantages of a semi-natural design.

Sticklebacks were fed a diet of frozen bloodworms three times a week, which was standardized (40g) across ponds. Fry were fed an additional diet of *daphnia* eggs and additional bloodworms as they matured. Feeding occurred three times a week at fixed times. In addition to the food provided, sticklebacks may have eaten small bugs in the water or on the water surface.

### 2.3 Behavioural assays

In semi-natural environments in the absence of a threat, sticklebacks tend to spread out and range over the entire area, which makes observation of individual behaviour difficult. Therefore, feeding times were used to attract all animals to a food cup 4.5cm in diameter containing bloodworms (Fig 1), which was attached to a long pole 10 cm from the ground and perforated so that sticklebacks had to tug bloodworms out one at a time. This increased the duration of foraging and prevented a small number of fish from eating all the food. Animals were allowed to feed until the food was gone. Feeding occurred three times a week (on Mondays, Wednesdays and Fridays), to motivate animals to approach and utilise the setup while still providing sufficient nutrition for development. An HD wide angle Hero GoPro camera was attached to the pole approximately 30cm above the food cup. Sticklebacks were trained to this setup for three weeks prior to the experiment (9 feedings).

Behavioural assays were conducted in 12 ponds in April 2012, at which time F1 were approximately one year old and not yet in their breeding state. Animals were presented with a novel object that obstructed access to the food cup. To this end, a transparent plastic wall was created around the food cup with a diameter of 30cm, which created three zones in the feeding area: close to the transparent wall, in the middle, or around the food cup. Access to the food cup could be obtained through nine holes of 3.8cm in diameter, placed in a 3x3 grid (Fig 1B and 1C). Placement of the novel object (along with the familiar feeding setup) in the water signified the start of the 30-minute experiment, which was video recorded in its entirety. After this, the obstacle was removed and animals were allowed regular feeding. Measurements in each pond were repeated 3 times a week for three consecutive weeks.

Video material was analysed for the first 10 individuals that entered the feeding area through the novel obstacle. Due to visual limitations inherent to the setup, it was not possible to keep track of more individuals, and in some ponds, no more than 10 individuals would enter at all for the duration of monitoring.

For each individual, a large set of behaviours was scored for every 10-second interval during 3 minutes (18 intervals). From these recordings, five behavioural measures were distilled to most closely represent relevant behavioural measures [46], see Table 1. Of the 652 individuals measured in this way, 417 were excluded as these fish could be tracked for less than two minutes (< 12 intervals). These fish hid behind conspecifics or the food cup, moved too fast to be distinguished from conspecifics, or left the setup, resulting in incomplete measurements. Of the remaining 235 individuals, 87% were tracked for the full 180 seconds (18 intervals) and 13% were tracked between 13 and 17 intervals. When only individuals who could be tracked for less than one minute (6 intervals) were excluded from analyses, results did not differ significantly. Although it is likely that some fish were measured repeatedly in subsequent feedings, the behaviour of the first 10 different fish to enter the setup at each moment of measuring still provides a reasonable representation of behavioural strategies expressed in these ponds.

### 2.4 Statistical Analyses

Differences between early-life environments (question 1) were analysed using linear mixed models with pond as a fixed factor, trial (number of the sample) as a random factor to correct for independent measurements, and date of testing as a covariate, as ponds were measured repeatedly (nine times) over the duration of the experiment, which caused possible habituation and learning effects in the sticklebacks. In addition, repeatabilities for each behaviour were calculated. Changes in behaviour over time were tested with a One-Way ANOVA. Relationships between behavioural measurements were investigated through multiple Pearson correlations (question 2). Effects of density on the behavioural measurements were tested through linear mixed models with density and date as covariates and trial as a random factor; similar analyses were performed for turbidity (question 3). All statistical analyses were performed using the statistical program SPSS 20.

### 2.5 Ethics

We adhered to the ‘Guidelines for the use of animals in research’ as published in Animal Behaviour (1991, 41, 183–186) and the Dutch laws on animal ethics standards. This research was approved by the IACUC-RuG conducted under DEC-code 6097A/B, as part of which animals well-being was evaluated daily by the animal care facilities of the University of Groningen. Water quality was checked monthly to ensure a stable and healthy water environment. Pond enrichments were available in the form of plastic plants for height, and plastic tubes for hideouts. Animals remained alive in the setup after the experiments.

## Results

### 3.1 Effects of rearing environment

**Fig 2:**
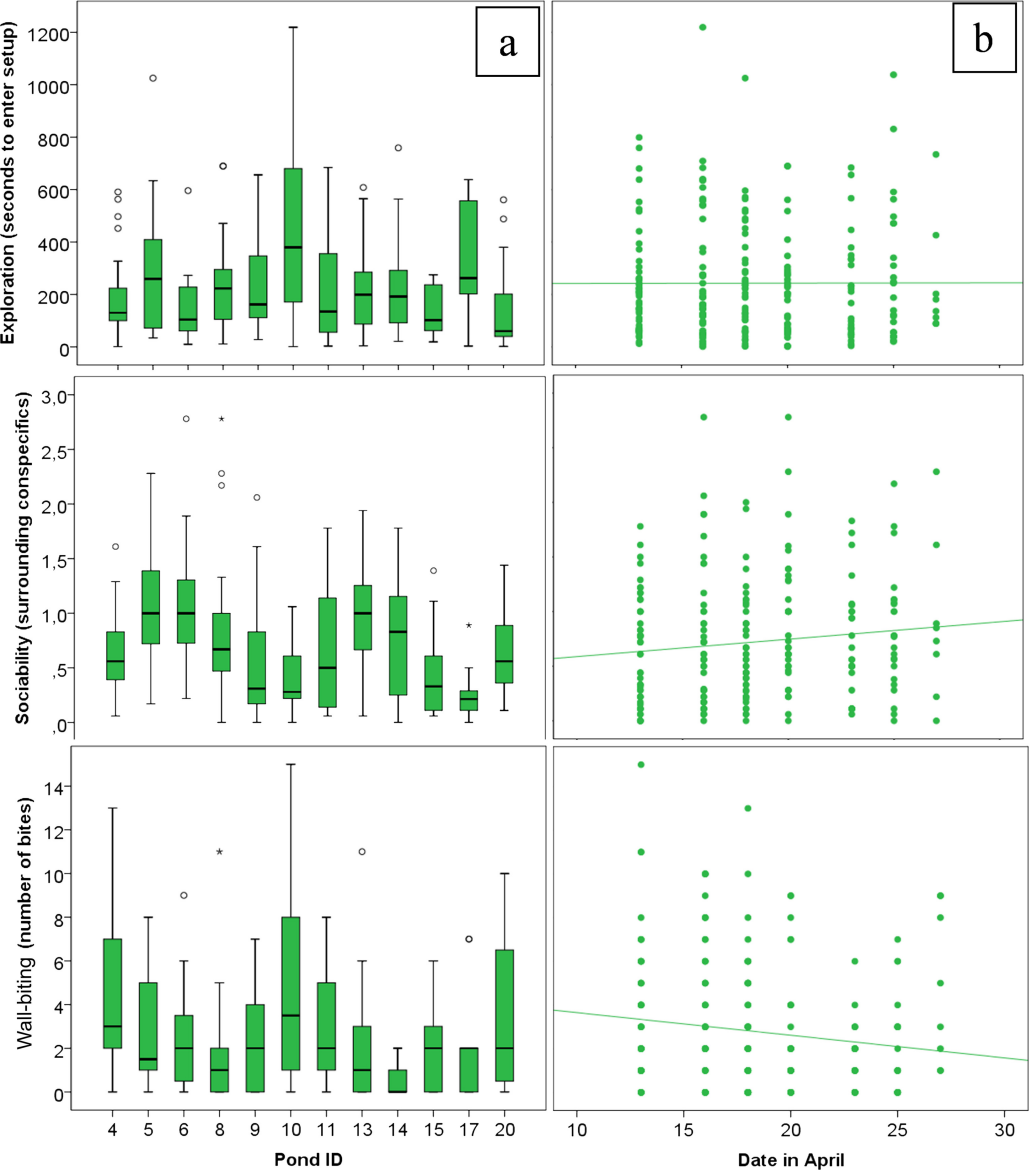
Spatial and temporal differences in coping behaviour. a. Exploration (F = 3.21, p<0.01), sociability (F = 3.33, p<0.01) and wall biting (F = 2.80, p<0.01) were affected by the early-life environment found in different ponds (boxplot with percentiles). Statistics from Table 2, model 1). b. Wall biting significantly decreased over the duration of the experiment (scatterplot with solid line indicating linear fit) (F = 7.00, p<0.1). N = 235 over 12 ponds.

Exploration, sociability and wall biting behaviours differed significantly between ponds with varying early-life environment, also when taking into account possible habituation and correcting for repeated experiments (Table 2, model 1). These were also the behaviours that were the most repeatable within ponds (Table 3), visually represented in Fig 2A. It is interesting to note here that ponds did not just differ in mean values, but also in within-pond variation (Fig 2A). Within the model testing for pond effects, wall biting was the only behaviour that significantly changed over time, decreasing over the course of the experiment (Table 3, model 1; Fig 2B). A trend was seen for changes in activity and sociability over the course of the month: activity decreased somewhat, whereas sociability increased (see Fig 2B).

**Table 2:** Mixed model analyses are given, including the random effects of date and trial, with F-statistic (pond, date, density, turbidity) or Wald Z (trial) and p-values for each effect included in the model. Significant effects are given in bold. Pond: 11 df, date: 1 df. Models for effects of density and turbidity on foraging gave warnings that the Hessian matrix was not always a positive definite. N = 235 over 12 ponds.

		Exploration		Activity		Foraging		Sociability		Wall biting	
Model	Effects	F / Z	p	F / Z	p	F / Z	p	F / Z	p	F / Z	p
**1**	*Pond*	3.21	**.01**	1.72	.11	0.67	.76	3.33	**.01**	2.80	**.01**
	*Date*	0.01	.95	3.72	.06	0.92	.34	2.61	.12	7.00	**.01**
	*Trial*	0.64	.53	1.11	.27	0.24	.81	1.62	.11	1.72	.09
**2**	*Density*	1.20	.28	0.01	.97	0.35	.56	12.71	**.01**	0.33	.57
	*Date*	0.01	.98	3.30	.08	1.11	.29	1.50	.23	3.00	.09
	*Trial*	2.13	**.03**	2.01	**.04**	10.77	.01	2.52	**.01**	2.89	**.01**
**3**	*Turbidity*	.13	.72	0.07	.79	0.01	.97	6.47	**.01**	0.21	.65
	*Date*	.01	.93	3.36	.07	1.28	.26	2.40	.13	2.85	.10
	*Trial*	2.2	**.03**	2.00	**.05**	10.77	**.01**	2.61	**.01**	2.85	**.01**

**Table 3:** Repeatability of behaviours within ponds. Df between groups: 11, within groups: 640, one-way ANOVA.

	MS within	MS between	F	p	R
**Exploration**	48940.301	592405.613	12.105	0.01	0.55
**Activity**	9.998	42.231	4.224	0.01	0.26
**Foraging**	44.121	115.360	2.615	0.03	0.15
**Sociability**	0.572	4.713	8.238	0.01	0.45
**Wall biting**	4.746	29.605	6.237	0.01	0.37

### 3.2 Behavioural correlations

Across all populations, behaviours from sticklebacks used in this experiment were all significantly correlated with each other. We calculated 10 correlations between behaviours, in which we did not correct for repeated measures for reasons outlined above. Individuals who entered the obstructed feeding area later also foraged less (r = -0.26), were more tolerant of others around them (r = 0.16), and displayed more wall-biting behaviour than individuals who entered sooner (r = 0.25). Wall biting was correlated with higher activity (r = 0.32), but also with lower sociability (r = -0.19) and lower foraging (r = -0.39), which matched visual observations of sticklebacks frantically swimming back and forth along the wall, subsequently spending less time on foraging or social interaction. Activity was negatively correlated with foraging (r = -0.24) and sociability (r = 0.26), which may be explained by the high activity levels of stressed animals. Animals who spent more time foraging also spent more time around conspecifics (r = 0.32).

Behavioural correlations between behaviours as reported above were not identical for all ponds. While in most ponds, activity was positively correlated with foraging and wall biting and exploration was negatively correlated with activity and foraging, in some ponds a pattern existed of correlations between sociability and exploration, foraging and wall biting. Due to the extent to which behaviours were correlated with each other, we were not able to test to what degree the between-pond differences in correlations were statistically significant.

### 3.3 Effects of group size and turbidity

The most obvious differences between the early-life environments consisted of the density in the pond (the number of fish present at the start of the experiment) and the algae content of the pond, measured here as turbidity. We modelled both density and turbidity to determine whether the pond effects found could be attributed to one of these environmental factors. Only sociability was significantly correlated with both the density (Table 2, model 2) and turbidity (Table 2, model 3) of the early-life environment. Sticklebacks from high density and high turbidity environments spent more time in the proximity of conspecifics (Table 4).

**Table 4:** Pearson’s correlations of behaviours with pond density and turbidity, as well as average values and SD. Significance at the 0.01 level is indicated by **. N = 253.

	Exploration	Activity	Foraging	Sociability	Wall biting	Mean	SD
Density	-.10	-.01	.05	.30**	.01	65	16
Turbidity	-.04	.03	-.01	.22**	-.06	.08	.02

## Discussion

### 4.1 Influence of mild early-life environmental changes on behaviours

The differences we found across ponds in the time it took sticklebacks to manoeuvre through the obstacle, in the amount of conspecifics they surrounded themselves with, and the amount of stress-related behaviour they showed, indicate that the early-life environment these sticklebacks experienced did–at least in some of the ponds–affect their ability to handle the human-induced disturbance of their feeding routine. As our study was exploratory rather than experimental, we cannot speculate about which ponds in particular encouraged increased adaptive behaviour, nor about which specific parameters were important in this. Nevertheless, these findings indicate that environmental conditions experienced by young animals during their development likely have an influence on the behaviour they show later in life–including their response to stressful situations, and the ability to adapt to human disturbance. Interestingly, only the behaviours exploration, sociability and wall biting differed significantly between environments. This suggests that whereas early-life environment is important both for the development of behaviour and for the sticklebacks’ responses to human-induced obstruction, some behaviours are especially sensitive to developmental influences. Given that social behaviour has been linked to stress responses [47], such sensitive behaviours may in fact all be reflections of animal difficulty in responding adaptively to an unfamiliar environment. Reduced exploration, increased anxiety and altered social behaviour as a group have been previously related to a single underlying cause [48].

Although it is always difficult to translate behaviours in artificial conditions into ecologically relevant concepts, after consideration of the correlations between behaviours, we feel that the behavioural measurements we used are an adequate representation of sticklebacks’ response to novel objects and challenging circumstances. What was measured as “exploration” may upon closer inspection of correlations better be called a variety of “boldness”, as individuals that entered the feeding area later generally also foraged less and changed zonation fewer times. A complication of measuring multiple behaviours as a response to a single situation is the attribution of causality. We cannot be confident which of the behaviours in our correlation matrix caused increases or decreases in the other variables, only that they are correlated. This creates a complex web of behaviours, which is difficult to interpret and raises multiple questions. For example, do fish who spend a lot of time wall-biting, sometimes considered a measure of stress-responsivity [34], group more with others because the company helps [47], or do fish surrounded by conspecifics get more stressed? Similarly, was higher density advantageous to development, or did the environment in these ponds provide a number of other advantages, leading to higher density? Even though this does not affect our main conclusion concerning the importance of early-life environment on adaptation, it does pose questions as to the underlying mechanism affecting the development of behaviour, and the interplay between abiotic environmental factors and sociality.

Compared to studies on rapid or intense environmental change [49], animals in our study experienced relatively mild and gradual changes, as the twelve environments differed only to the extent that they could naturally diversify over the course of one year. This suggests that changes do not need to be rapid in onset, nor large in magnitude, nor novel in quality [8], to still affect animals’ adaptive responses. Gradual, naturally occurring changes over the span of a year already seem to have provided enough variation that wall-biting behaviours in response to a human-induced obstacle differed between semi-natural populations. Furthermore, the early-life environment had a significant impact even though conditions during ontogeny seasonally varied. Sticklebacks in this experiment experienced warm summer days, algae blooms, rain, frost, and the changing of day length, all of which can be assumed to impact their behaviour. Yet despite these fluctuations, which affected each of the ponds equally, small differences between ponds still had an effect on exploratory and feeding behaviours. Natural fluctuations may under certain conditions already affect whether or not a population of animals will be successful in dealing with environmental disruption, and should not be underestimated when attempting to predict population responses to change [44].

### 4.2 Discussion of methods

A strength of this study is the use of semi-natural ponds and outdoor-dwelling individuals, which allowed us to measure behaviour that is much more closely related to natural values [50] than behaviour measured under laboratory conditions. Sticklebacks in each pond lived together since birth, during which time they established territories and natural social interactions that were left entirely undisturbed. Behaviours were measured in their native environments, without exposure to relocation, isolation or added experimental stress, and within a familiar social group. Since sticklebacks, as highly social animals, are strongly affected in their exploration and foraging behaviour by the presence of conspecifics from their own social group [28], the behaviours described in this study are likely to be more representative of conditions in the wild than would be the case in a lab study. As a result of the semi-natural setting, however, we inevitably opened ourselves up for difficulties in measuring the resulting behaviour. We excluded over 400 individuals from analyses because turbidity of the water and low contrast with the surroundings made it impossible to track them long enough to get a reliable indication of their behaviour, even when using high-quality cameras. In addition, the statistical assumption of random sampling could not be confirmed, and it is quite possible that certain fish always arrived first into the experimental setup, which may have biased pond averages towards a greater degree of explorative behaviour. However, we did control for density in the analyses and found no effect except on sociality (see Table 4), which reassures us that effects of repeated measures due to density effects are negligible.

### 4.3 Implications and Future directions

While, this study was not aimed at determining which environmental factors exactly contributed to the behavioural differences between early-life environments, or which developmental mechanisms contributed to these differences, it does illustrate that behaviours measured in one population, in a particular context, can differ importantly not only from other populations and other contexts, but even from near identical populations, in near identical circumstances. While ecologically the circumstances between our ponds differed, perhaps in important ways, these differences are minute compared to the differences found in existing natural habitats. From our findings, we can extrapolate that small environmental differences may have pronounced effect on resultant population behaviours.

Moreover, we can speculate that small environmental variations during development may have important consequences for animal behaviour that are difficult to predict from the starting conditions, suggesting that the ability of natural populations to respond adaptively to environmental change and human interference is flexible and context-dependent.

We caution researchers in the field of conservation biology that population-level responses to environmental change may diverge from predicted values based on earlier work, depending on conditions during ontogeny and the inherent level of plasticity of the species. An important next step is to address the underlying mechanisms in order to better understand what characteristic(s) in the environment cause the differences in behaviour between environments. Is there a simple way to predict environmental factors that facilitate an adaptive behavioural response, such as maintaining a certain population size or environmental stability, or would environmental effects have to be assessed on a case-to-case basis?

## Supporting Information

S1 Fig (SAV)

## References

[1] Boyce WT, Ellis BJ. Biological Sensitivity to Context: I. An Evolutionary–developmental Theory of the Origins and Functions of Stress Reactivity. Dev Psychopathol. 2005;17: 271–301. 10.1017/S0954579405050145 16761546

[2] Bradshaw WE, Holzapfel CM. Light, Time, and the Physiology of Biotic Response to Rapid Climate Change in Animals. Annu Rev Physiol. 2010;72: 147–166. 10.1146/annurev-physiol-021909-135837 20148671

[3] Storey KD, Tanino K. Temperature Adaptation in a Changing Climate: Nature at Risk. CABI; 2012.

[4] Fletcher RJ, Sieving KE. Social-Information use in Heterogeneous Landscapes: A Prospectus. The Condor. 2010;112: 225–234. 10.1525/cond.2010.090236

[5] Abellán P, Millán A, Ribera I. Parallel habitat-driven differences in the phylogeographical structure of two independent lineages of Mediterranean saline water beetles. Mol Ecol. 2009;18: 3885–3902. 10.1111/j.1365-294X.2009.04319.x 19702753

[6] Davis MB. Range Shifts and Adaptive Responses to Quaternary Climate Change. Science. 2001;292: 673–679. 10.1126/science.292.5517.673 11326089

[7] Lande R. Adaptation to an extraordinary environment by evolution of phenotypic plasticity and genetic assimilation. J Evol Biol. 2009;22: 1435–1446. 10.1111/j.1420-9101.2009.01754.x 19467134

[8] Langenhof MBW, Komdeur J. Coping with Change: A Closer Look at the Underlying Attributes of Change and the Individual Response to Unstable Environments. Sustainability. 2013;5: 1764–1788. 10.3390/su5051764

[9] Garduño-Paz MV, Couderc S, Adams CE. Habitat complexity modulates phenotype expression through developmental plasticity in the threespine stickleback. Biol J Linn Soc. 2010;100: 407–413. 10.1111/j.1095-8312.2010.01423.x

[10] Hofmann HA, Benson ME, Fernald RD. Social status regulates growth rate: Consequences for life-history strategies. Proc Natl Acad Sci. 1999;96: 14171–14176. 10.1073/pnas.96.24.14171 10570217 PMC24209

[11] Calsbeek R. Experimental evidence that competition and habitat use shape the individual fitness surface. J Evol Biol. 2009;22: 97–108. 10.1111/j.1420-9101.2008.01625.x 19120813

[12] Gracceva G, Koolhaas JM, Groothuis TGG. Does the early social environment affect structure and consistency of personality in wild-type male rats? Dev Psychobiol. 2011;53: 614–623. 10.1002/dev.20586 21761410

[13] Gonda A, Herczeg G, Merilä J. Habitat-dependent and -independent plastic responses to social environment in the nine-spined stickleback (Pungitius pungitius) brain. Proc R Soc B Biol Sci. 2009; rspb.2009.0026. 10.1098/rspb.2009.0026 PMC2677246 19324759

[14] Bell AM, Dingemanse NJ, Hankison SJ, Langenhof MBW, Rollins K. Early exposure to nonlethal predation risk by size-selective predators increases somatic growth and decreases size at adulthood in threespined sticklebacks. J Evol Biol. 2011;24: 943–953. 10.1111/j.1420-9101.2011.02247.x 21375647 PMC3968075

[15] Groothuis TGG, Müller W, von Engelhardt N, Carere C, Eising C. Maternal hormones as a tool to adjust offspring phenotype in avian species. Neurosci Biobehav Rev. 2005;29: 329–352. 10.1016/j.neubiorev.2004.12.002 15811503

[16] McGowan PO, Sasaki A, D’Alessio AC, Dymov S, Labonté B, Szyf M, et al Epigenetic regulation of the glucocorticoid receptor in human brain associates with childhood abuse. Nat Neurosci. 2009;12: 342–348. 10.1038/nn.2270 19234457 PMC2944040

[17] Weaver ICG, Cervoni N, Champagne FA, D’Alessio AC, Sharma S, Seckl JR, et al Epigenetic programming by maternal behavior. Nat Neurosci. 2004;7: 847–854. 10.1038/nn1276 15220929

[18] Laland KN, Uller T, Feldman MW, Sterelny K, Müller GB, Moczek A, et al The extended evolutionary synthesis: its structure, assumptions and predictions. Proc R Soc B. 2015;282: 20151019 10.1098/rspb.2015.1019 26246559 PMC4632619

[19] Grether GF. Environmental Change, Phenotypic Plasticity, and Genetic Compensation. Am Nat. 2005;166: E115–E123. 16224697 10.1086/432023

[20] Richter S, Kipfer T, Wohlgemuth T, Guerrero CC, Ghazoul J, Moser B. Phenotypic plasticity facilitates resistance to climate change in a highly variable environment. Oecologia. 2012;169: 269–279. 10.1007/s00442-011-2191-x 22081261

[21] Sih A, Ferrari MCO, Harris DJ. Evolution and behavioural responses to human-induced rapid environmental change. Evol Appl. 2011;4: 367–387. 10.1111/j.1752-4571.2010.00166.x 25567979 PMC3352552

[22] Tuomainen U, Candolin U. Behavioural responses to human-induced environmental change. Biol Rev. 2011;86: 640–657. 10.1111/j.1469-185X.2010.00164.x 20977599

[23] Crooks KR, Burdett CL, Theobald DM, Rondinini C, Boitani L. Global patterns of fragmentation and connectivity of mammalian carnivore habitat. Philos Trans R Soc B Biol Sci. 2011;366: 2642–2651. 10.1098/rstb.2011.0120 PMC3140740 21844043

[24] Jenssen BM. Endocrine-Disrupting Chemicals and Climate Change: A Worst-Case Combination for Arctic Marine Mammals and Seabirds? Environ Health Perspect. 2005;114: 76–80. 10.1289/ehp.8057 PMC1874189 16818250

[25] Nussey DH, Wilson AJ, Brommer JE. The evolutionary ecology of individual phenotypic plasticity in wild populations. J Evol Biol. 2007;20: 831–844. 10.1111/j.1420-9101.2007.01300.x 17465894

[26] Harcourt JL, Biau S, Johnstone R, Manica A. Boldness and Information Use in Three-Spined Sticklebacks. Ethology. 2010;116: 440–447. 10.1111/j.1439-0310.2010.01757.x

[27] Bjærke O, Østbye K, Lampe HM, Vøllestad LA. Covariation in shape and foraging behaviour in lateral plate morphs in the three-spined stickleback. Ecol Freshw Fish. 2010;19: 249–256. 10.1111/j.1600-0633.2010.00409.x

[28] Huntingford FA, Ruiz-Gomez ML. Three-spined sticklebacks Gasterosteus aculeatus as a model for exploring behavioural biology. J Fish Biol. 2009;75: 1943–1976. 10.1111/j.1095-8649.2009.02420.x 20738667

[29] Dingemanse NJ, Bouwman KM, van de Pol M, van Overveld T, Patrick SC, Matthysen E, et al Variation in personality and behavioural plasticity across four populations of the great tit Parus major. J Anim Ecol. 2012;81: 116–126. 10.1111/j.1365-2656.2011.01877.x 21692798

[30] Minderman J, Reid JM, Evans PGH, Whittingham MJ. Personality traits in wild starlings: exploration behavior and environmental sensitivity. Behav Ecol. 2009;20: 830–837. 10.1093/beheco/arp067

[31] Koolhaas JM, Bartolomucci A, Buwalda B, de Boer SF, Flügge G, Korte SM, et al Stress revisited: A critical evaluation of the stress concept. Neurosci Biobehav Rev. 2011;35: 1291–1301. 10.1016/j.neubiorev.2011.02.003 21316391

[32] Anisman H, Zaharia MD, Meaney MJ, Merali Z. Do early-life events permanently alter behavioral and hormonal responses to stressors? Int J Dev Neurosci. 1998;16: 149–164. 10.1016/S0736-5748(98)00025-2 9785112

[33] Bell A, Henderson L, Huntingford F. Behavioral and respiratory responses to stressors in multiple populations of three-spined sticklebacks that differ in predation pressure. J Comp Physiol [B]. 2010;180: 211–220. 10.1007/s00360-009-0395-8 PMC4899084 19705129

[34] Stamps J, Groothuis TGG. The development of animal personality: relevance, concepts and perspectives. Biol Rev. 2010;85: 301–325. 10.1111/j.1469-185X.2009.00103.x 19961473

[35] Dingemanse NJ, Wright J, Kazem AJN, Thomas DK, Hickling R, Dawnay N. Behavioural syndromes differ predictably between 12 populations of three-spined stickleback. J Anim Ecol. 2007;76: 1128–1138. 10.1111/j.1365-2656.2007.01284.x 17922709

[36] Bell A m. Behavioural differences between individuals and two populations of stickleback (Gasterosteus aculeatus). J Evol Biol. 2005;18: 464–473. 10.1111/j.1420-9101.2004.00817.x 15715852

[37] Bell AM, Sih A. Exposure to predation generates personality in threespined sticklebacks (Gasterosteus aculeatus). Ecol Lett. 2007;10: 828–834. 10.1111/j.1461-0248.2007.01081.x 17663716

[38] Herczeg G, Gonda A, Merilä J. The social cost of shoaling covaries with predation risk in nine-spined stickleback, Pungitius pungitius, populations. Anim Behav. 2009;77: 575–580. 10.1016/j.anbehav.2008.10.023

[39] Naguib M, Flörcke C, van Oers K. Effects of social conditions during early development on stress response and personality traits in great tits (Parus major). Dev Psychobiol. 2011;53: 592–600. 10.1002/dev.20533 21365640

[40] Vlieger L, Candolin U. How not to be seen: does eutrophication influence three-spined stickleback Gasterosteus aculeatus sneaking behaviour? J Fish Biol. 2009;75: 2163–2174. 10.1111/j.1095-8649.2009.02403.x 20738680

[41] Heuschele J, Mannerla M, Gienapp P, Candolin U. Environment-dependent use of mate choice cues in sticklebacks. Behav Ecol. 2009;20: 1223–1227. 10.1093/beheco/arp123

[42] Kraak SBM, Bakker TCM, Mundwiler B. Sexual selection in sticklebacks in the field: correlates of reproductive, mating, and paternal success. Behav Ecol. 1999;10: 696–706. 10.1093/beheco/10.6.696

[43] Bradbury RB, Payne RJH, Wilson JD, Krebs JR. Predicting population responses to resource management. Trends Ecol Evol. 2001;16: 440–445. 10.1016/S0169-5347(01)02189-9

[44] Keith DA, Akçakaya HR, Thuiller W, Midgley GF, Pearson RG, Phillips SJ, et al Predicting extinction risks under climate change: coupling stochastic population models with dynamic bioclimatic habitat models. Biol Lett. 2008;4: 560–563. 10.1098/rsbl.2008.0049 18664424 PMC2610061

[45] Ostlund-Nilsson S, Mayer I, Huntingford FA. Biology of the Three-Spined Stickleback. CRC Press; 2006.

[46] Bell AM, Stamps JA. Development of behavioural differences between individuals and populations of sticklebacks, Gasterosteus aculeatus. Anim Behav. 2004;68: 1339–1348. 10.1016/j.anbehav.2004.05.007

[47] Scheiber IBR, Kotrschal K, Weiß BM. Benefits of family reunions: Social support in secondary greylag goose families. Horm Behav. 2009;55: 133–138. 10.1016/j.yhbeh.2008.09.006 18848947 PMC3182547

[48] Balemans MCM, Huibers MMH, Eikelenboom NWD, Kuipers AJ, van Summeren RCJ, Pijpers MMCA, et al Reduced exploration, increased anxiety, and altered social behavior: Autistic-like features of euchromatin histone methyltransferase 1 heterozygous knockout mice. Behav Brain Res. 2010;208: 47–55. 10.1016/j.bbr.2009.11.008 19896504

[49] Hecky RE, Mugidde R, Ramlal PS, Talbot MR, Kling GW. Multiple stressors cause rapid ecosystem change in Lake Victoria. Freshw Biol. 2010;55: 19–42. 10.1111/j.1365-2427.2009.02374.x

[50] Sweeney K, Gadd RDH, Hess ZL, McDermott DR, MacDonald L, Cotter P, et al Assessing the Effects of Rearing Environment, Natural Selection, and Developmental Stage on the Emergence of a Behavioral Syndrome. Ethology. 2013;119: 436–447. 10.1111/eth.12081

[51] Day T, McPhail JD. The effect of behavioural and morphological plasticity on foraging efficiency in the threespine stickleback (&lt;i&gt;Gasterosteus&lt;/i&gt; sp.). Oecologia. 1996;108: 380–388. 10.1007/BF00334665 28307853

[52] Pike TW, Laland KN. Conformist learning in nine-spined sticklebacks’ foraging decisions. Biol Lett. 2010;6: 466–468. 10.1098/rsbl.2009.1014 20129948 PMC2936200

